# Selection of den sites and chronology of denning by black bears in the eastern Sierra Nevada and western Great Basin

**DOI:** 10.1002/ece3.11689

**Published:** 2024-07-10

**Authors:** Morgan E. Long, Kelley M. Stewart, Kevin T. Shoemaker, Heather Reich, Carl W. Lackey, Jon P. Beckmann

**Affiliations:** ^1^ Ecology Evolution and Conservation Biology Graduate Program and Department of Natural Resources and Environmental Science University of Nevada Reno Nevada USA; ^2^ Nevada Department of Wildlife Reno Nevada USA; ^3^ Wildlife Conservation Society Bozeman Montana USA; ^4^ Present address: Kansas Department of Wildlife and Parks Pratt Kansas USA

**Keywords:** American black bears, den‐site selection, Great Basin, hibernation, Sierra Nevada, *Ursus americanus*

## Abstract

Use of dens during winter is an important strategy for American black bears (*Ursus americanus*) for both energy conservation and reproduction; and occupancy of suitable den sites has implications for reproductive fitness. Denning strategies may change as a result of changing climatic conditions and habitat loss. Black bears occupy arid environments in the eastern Sierra Nevada and the western ranges of the Great Basin Ecosystem. Our objectives were to identify: (1) which physical characteristics of habitat influenced selection of den sites at multiple spatial scales and (2) which environmental factors influenced timing of entrance and exit of dens by females and males. We evaluated selection of den sites by black bears at three spatial scales (300, 1000, and 4000 m) from 2011 to 2022. Terrain ruggedness was important for selection of den sites at all spatial scales. Within a 300‐m buffer from the den, bears selected den sites with rugged terrain, lower horizontal visibility, and greater canopy cover, resulting in more concealment and protection than that of the surrounding environment. Within 1000‐ and 4000‐m buffers around each den, bears selected den sites with rugged terrain, northern aspects, and steep slopes. At the 4000‐m scale, we observed interactions between sex with slope and distance to roads; females selected den sites on steeper slopes and closer to roads than did males. Females remained in the dens longer than males by entering earlier in the autumn and exiting later in the spring. Male bears exited their dens earlier with increasing consecutive days above freezing temperatures, but that relationship was weak for females. Knowing what characteristics are important for selection of den sites, and influence timing of denning, will be important for understanding how shifting climatic patterns will affect bears, particularly in arid environments that may be prone to wider fluctuations in climatic drivers of denning in the future.

## Graphical abstract

Selection of appropriate den sites for hibernation is an important strategy for American black bears (*Ursus americanus*) for both energy conservation and reproduction, because parturition occurs while females are occupying their dens. Our objectives were to identify which physical characteristics of the habitat influenced selection of den sites and what environmental factors influenced timing of entrance and exit of dens between females and males. Bears selected dens with high concealment, rugged terrain, and steep slopes. Males exited dens sooner than females with increasing number of consecutive days above freezing.

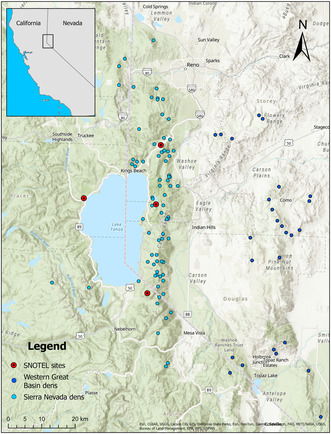

## 1 INTRODUCTION

Multiple species that experience environments with seasonal fluctuations in weather and availability of food have evolved strategies to survive in those conditions. Those strategies include moving to more mild climate conditions though migration (Berger, 2004; Hayes, 1995), seasonal breeding (Fuglei & Ims, 2008), or through periods of inactivity using torpor or hibernation (Bieber et al., 2012; Fowler et al., 2021; Hellgren, 1998; Humphries et al., 2003). Hibernation is described as suppression of body temperature and metabolism for extended periods of time, which is coincident with use of dens by many species including members of the family Ursidae (Fowler et al., 2021; Hellgren, 1998; Melvin & Andrews, 2009). Use of dens over winter is a strategy used by many species of mammals to survive seasons of extreme weather and food scarcity (Humphries et al., 2003). Broadly defined, a den is a secure area or hideout where an animal spends time and is primarily used for rest, sleep (including torpor or hibernation), or reproduction (Davis et al., 2012; Libel et al., 2011; Robitaille et al., 2020). Dens used by bears (*Ursus* spp.) are areas where bears spend the winter, and can range from a hollow tree, under a rock or pile of rocks, under a shrub, to a shallow depression in the ground, bears may excavate or modify their dens or use the existing structure (Bard & Cain III, 2020; Beecham et al., 1983; Pelton et al., 1980; Ryan & Vaughan, 2004). A den site describes characteristics in immediate area around the location of the den. Denning is an energetically advantageous strategy when the net costs of building and using a den are lower than the cost of remaining active during the same time period (Fowler et al., 2021; Humphries et al., 2003). Dens are used to protect both adults and young; and individuals select and use dens for thermal stability, energy efficiency, shelter from weather, protection during parturition, and protection from inter‐ and intraspecific predation on adults and young. Dens may be located in close proximity to sources of food that would be available upon exit (Boutros et al., 2007; Laurenson, 1994).

All species of bears in North America, including American black bears (*Ursus americanus*), polar bears (*Ursus maritimus*), and brown bears (*Ursus arctos*) use dens during winter for either hibernation, parturition, or both (Hellgren, 1998; Nelson et al., 1980). The time period when bears are within dens is not only important for energy conservation, but also for reproduction because parturition occurs and females begin to care for offspring while occupying dens (Anderson et al., 2012; Bard & Cain III, 2020; Pelton et al., 1980). Bears cannot readily flee dens if disturbed without substantial energetic costs, and disturbance also can result in lowered body condition of adults and reduced survival of young (Baldwin & Bender, 2008). Dens that were more energetically efficient resulted in brown bears emerging from dens in better body condition than those occupying dens that were less energy efficient (Shiratsuru et al., 2020). Time spent in dens during winter may vary across a species range or annually with fluctuations in climate and availability of resources (Amspacher et al., 2023). Therefore, selection of an appropriate den site is important for population growth or stability as well as survival and recruitment of young. Previous work has suggested that the type of den that bears selected was consistent with the type of cover that was most common in the surrounding area (Baldwin & Bender, 2008; Bard & Cain III, 2020; Beecham et al., 1983; Fowler et al., 2019; Schafer et al., 2018).

Timing of entry and exit from dens is also an important aspect of den occupation, and could affect both body condition of bears and reproductive success (López‐Alfaro et al., 2013). Previous work has demonstrated differences in chronology of den entrance and exit between females and males; generally, males enter dens later and remain in the den for a shorter amount of time than do females (Beckmann & Berger, 2003; Fowler et al., 2019; Waller et al., 2012). Females have higher reproductive demand than males because of pregnancy and parturition, and therefore may enter the den earlier and exit later because of those demands (Doan‐Crider & Hellgren, 1996; Friebe et al., 2014). Johnson et al. (2017) reported that female bears with dependent young entered dens earlier than females without young. Body condition of female bears can influence survival of young, so hibernation to conserve energy until parturition is especially important for females (Noyce & Garshelis, 1994). Previous work has found timing of entrance and exit of dens to be variable (Bard & Cain III, 2020; Beecham et al., 1983; Fowler et al., 2019; Waller et al., 2012), and the period of hibernation can range from 0 to 212 days depending on seasonal food availability and winter severity (Fowler et al., 2021). Timing of entrance specifically has been correlated with decreasing abundance of forage plants (Fowler et al., 2019; Pigeon, Stenhouse, & Cote, 2016). While a specific environmental trigger or initiation of den entrance is not fully known, lower autumn temperatures, lower food availability, presence of snow, and incoming storms have been correlated with bears entering dens (Baldwin & Bender, 2010; Fowler et al., 2019; Friebe et al., 2001; Schooley et al., 1994). Food subsidies from areas of human habitation may also affect denning by providing consistent food sources throughout the year, even causing some individuals to forego denning entirely (Amspacher et al., 2023; Krofel et al., 2017). Previous research has also shown temperature to influence exit from dens, and movement away from dens following emergence may in part be driven by warming temperatures (Fowler et al., 2019; Gonzalez‐Bernardo, Giulia, et al., 2020; Johnson et al., 2017; Waller et al., 2012).

The climate, aridity, and topography of the eastern Sierra Nevada and western Great Basin as well as proximity to humans may cause patterns of denning to differ from other areas where denning behavior had previously been studied. The objective of this study was to investigate selection of den sites and timing of entrance into and exit from dens by black bears in the eastern Sierra Nevada and western Great Basin of Nevada. We hypothesized that bears would select primarily rock or tree dens across our study areas, because large trees are common in the Sierra Nevada and rock piles are common in both the Sierra Nevada and Western Great Basin. We hypothesized that den sites would have high concealment and would be located far from human impacted areas and sources of disturbance. Based on that hypothesis, we predicted that bears would select dens with higher concealment (low visibility) and greater distance to the nearest road than was generally available in the study area. Additionally, we hypothesized that females and males would differ in type of den selected, such that females would select den types that provide more protection than dens selected by males. In investigating chronology, we hypothesized that mean entrance and exit dates would differ among sexes and that females would enter dens earlier and exit later than males. We hypothesized that entrance and exit would be correlated with temperature and the presence or absence of snow preceding entrance or exit from dens. We predicted that earlier entrance dates within the dataset would be correlated with cooler temperatures and deeper snow in autumn, and that earlier exit dates would be correlated with warmer spring temperatures and lower snow depth.

## 2 MATERIALS AND METHODS

### 2.1 Study area

**FIGURE 1:**
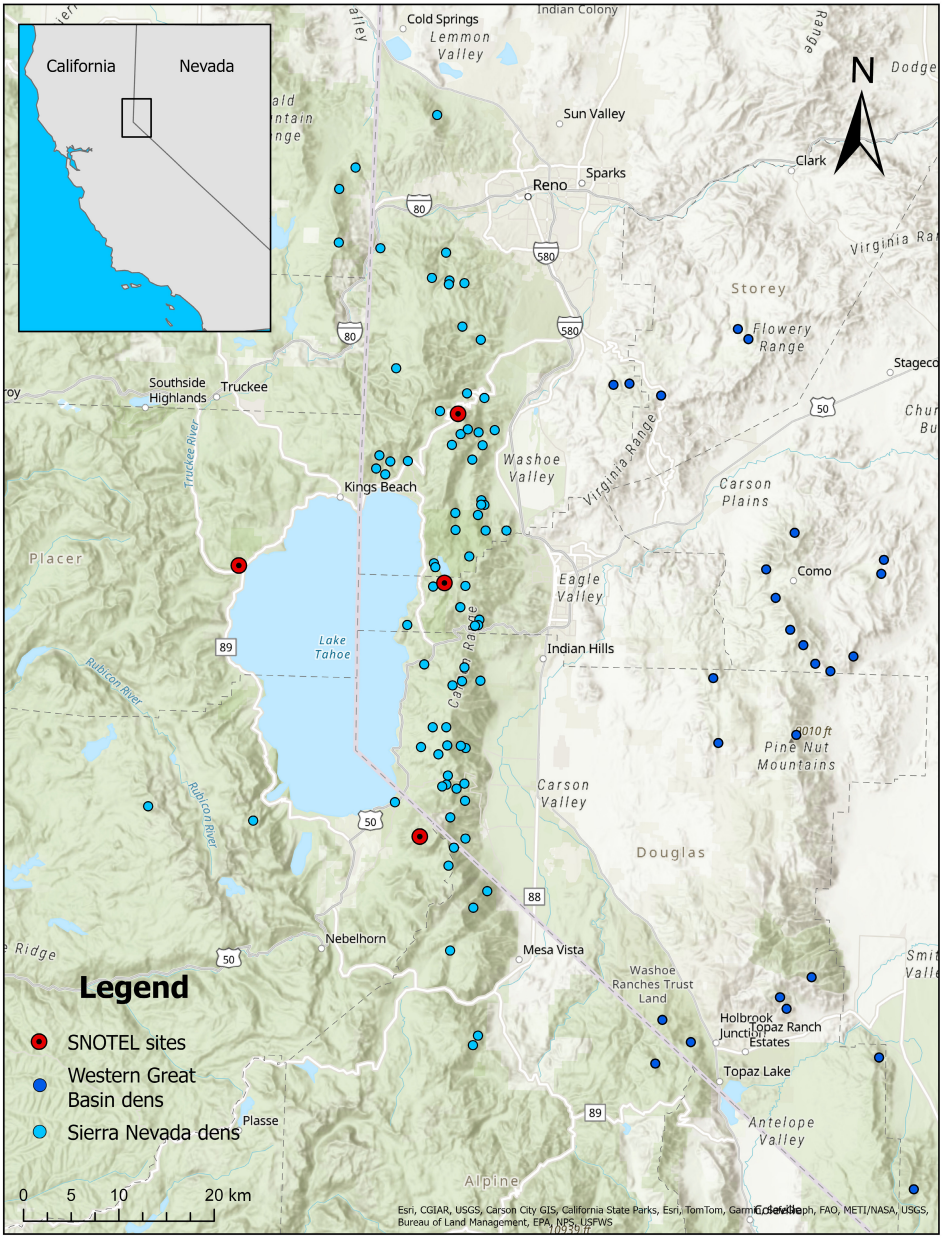
Map of 116 black bear den sites in the eastern Sierra Nevada (light blue points) and western Great Basin (dark blue points) identified from 2011 to 2022, and selected Snowpack Telemetry (SNOTEL) sites within the eastern Sierra Nevada.

The study area consists of two eco‐regions in northwest Nevada, USA: the Sierra Nevada Mountains east of Lake Tahoe (eastern Sierra Nevada) and the western Great Basin (Figure 1). This study area is unique in that it is one of the driest regions occupied by black bears, and there is a large wildland–urban interface, meaning bears may forage in human occupied areas, but den in wildlands (Beckmann & Berger, 2003; van Manen et al., 2019). While black bears may now be found across much of Nevada (Lackey et al., 2013), our study area includes the mountain regions where bears are most common: The Carson Range in the eastern Sierra Nevada, the Pine Nut Mountains, and the Virginia Range in the western Great Basin, as well as surrounding areas and nearby basins (Figure 1). Elevations range from a maximum of 3316 m in the Carson Range, 2397 m in the Virginia Range, and 2882 m in the Pine Nut Mountains, to minimum elevations within the basins around 1200 m.

Land cover differs between these eco‐regions: the eastern Sierra Nevada is characterized by tree species, which includes ponderosa pine (*Pinus ponderosa*), Jeffrey pine (*Pinus jeffreyi*), lodgepole pine (*Pinus contorta*), and mountain hemlock (*Tsuga mertensiana*) (Andreasen et al., 2021). Understory communities in the eastern Sierra Nevada are often made up of sagebrush (*Artemisia tridentata*), tobacco brush (*Cercocarpus velutinus*), and rabbitbrush (*Ericameria* spp.) (Andreasen et al., 2021). The Great Basin predominantly consists of mixed sagebrush (*A. tridentata*) and woodlands dominated by single‐leaf pinyon pine (*Pinus monophylla*) and Utah‐juniper (*Juniperus osteosperma*) (Andreasen et al., 2021; Beckmann & Berger, 2003; Lackey et al., 2013; Wynn‐Grant et al., 2018). At visited den sites, we identified plants in the field, and taxonomy was derived from the U.S. Department of Agriculture (USDA) PLANTS database (USDA NRCS, 2023). The most common shrub was manzanita (*Arctostaphylos patula*). Other common plants found near den sites included: Ferns (*Pteridophyta* group), woolly mules‐ear (*Wyethia mollis*), woods' rose (*Rosa woodsii*), Sierra currant (*Ribes nevadense*), Douglas' sagewort (*Artemisia douglasiana*), antelope bitterbrush (*Purshia tridentata*), winterfat (*Krascheninnikovia lanata*), and big sagebrush. Trees surrounding den sites were predominantly Jeffrey pine, pinyon pine, and juniper.

### 2.2 Data collection

Our dataset consisted of den‐site locations of black bears from 2011 to 2022, which was provided by the Nevada Department of Wildlife (NDOW). A total of 116 den sites were identified over that time frame and NDOW biologists visited 76 of those sites from 2011 to 2022 to confirm location, presence and sex of the bear, and type of den. Locations of den sites were determined by NDOW using GPS (Global Positioning System) collared bears and were identified as a den either when GPS locations formed a cluster of spatial points over the winter, when satellite transmissions or loss of reception from the collar indicated that a bear had quit moving, or data from spring indicated that the bear had exited the den. We removed five dens from our study because they occurred underneath human homes, and bears were encouraged to vacate the structure by NDOW biologists.

We grouped dens into five types: “rock,” “tree,” “excavated,” “exposed,” or “other.” Bears in our study either used an existing structure such as a cavity under rocks or inside a hollow tree, but there were also indications that bears modified those sites or excavated new sites. We defined rock dens as a den underneath a single rock or pile of rocks with a cavity below. We defined tree dens as a standing tree that was dug out underneath by the bear or was already hollow. Dens excavated by bears had no preexisting structure, but were made up of shallow holes in the ground or used brush piles. Exposed dens were areas with no excavation around the den site and no direct use of surrounding trees or rocks, although exposed dens were often found at the base of trees. We also included a group of five dens, which were categorized as “other.” Those dens occurred in anthropogenic structures (not houses), which included culverts under roads and abandoned mines.

In addition to the 76 dens visited by NDOW biologists from 2011 to 2022, we visited 26 additional den sites to collect data on vegetation and habitat structure, predominantly in the Carson Range of the Sierra Nevada from August to December 2022. At those 26 sites, we measured characteristics from the den opening within a 15‐m diameter circular plot around the den and two randomly generated sites within a 300‐m radius from the den (Bard & Cain III, 2020). Those characteristics included: dominant vegetation type, ground cover (%), number of trees, tree cover (%), horizontal visibility (%), and diameter at breast height (DBH) of the two largest trees to generally characterize the size of trees at each site (Tables 1 and 2). The 26 dens selected for field data collection were randomly selected from the full set of 116 dens, but we also considered feasibility and safety of accessing those sites. We visually estimated the dominant vegetation type as one of the following categories: bare ground, shrub (denoting family of shrub), trees, or leaf litter. We also visually estimated percent cover of those vegetation categories, including shrub species. For measurements involving estimations, the same researcher made the estimates each time to ensure consistency of measurements among sites. We measured tree cover (%) at five sites within each plot using a spherical convex densiometer (Lemon, 1956) at the center of the plot facing north and then at the edge of the plot along each of the four cardinal directions facing the center (Bard & Cain III, 2020; Pigeon, Cote, & Stenhouse, 2016). We measured horizontal visibility using a 1‐m tall cylinder with a 33‐cm radius (Bard & Cain III, 2020; Ordiz et al., 2009). We placed the cylinder at the center of the plot and recorded the percentage of the cylinder that was visible from each cardinal direction at the edge of the plot observed from 1 m above the ground. We calculated a minimum distance of total concealment, by measuring the minimum distance away from the den in which the cylinder was no longer visible (Bard & Cain III, 2020).

**TABLE 1:** Summary statistics, means ± standard deviations, of geospatial landscape‐level characteristics and local characteristics measured in the field at den sites of black bears (*n* = 26) and random sites within a 300‐m buffer (*n* = 52), and remotely at den sites (*n* = 116) and random sites (*n* = 9280, 17,400) within 300‐, 1000‐ and 4000‐m buffers.

Variable	Den sites ($\underline{x}$ ± SD)	Random sites ($\underline{x}$ ± SD)
Den sites and random points at 300‐m scale with field characteristics		
Distance to road (m)	426 ± 429.1	448 ± 432.5
Elevation (m)	2148.0 ± 236.71	2149.4 ± 243.02
Horizontal visibility (%)	37 ± 20	70 ± 25
Tree cover (%)	36 ± 29.4	33 ± 27.5
Slope (°)	16.9 ± 6.16	16.5 ± 6.24
Aspect (°)	142.8 ± 91.6	136.7 ± 95.12
Bare ground (%)	49 ± 27.8	53 ± 22.2
Ruggedness	0.0012 ± 0.00122	0.0008 ± 0.0006
Den sites and random points at 300‐m scale		
Distance to road (m)	878 ± 913.1	875 ± 910.2
Elevation (m)	2190 ± 329.7	2184 ± 328.1
Tree cover (%)	29 ± 27.1	29 ± 26.7
Slope (°)	19 ± 8.2	18 ± 8.2
Aspect (°)	156 ± 95.2	150 ± 96.8
Ruggedness	0.0011 ± 0.0015	0.0008 ± 0.0012
Den sites and random points at 1000‐m scale		
Distance to road (m)	878 ± 913.1	840 ± 919.0
Elevation (m)	2190 ± 329.7	2178 ± 329.6
Tree cover (%)	29 ± 27.1	29 ± 27.1
Slope (°)	19 ± 8.2	17 ± 8.0
Aspect (°)	156 ± 95.2	153 ± 99.3
Ruggedness	0.0011 ± 0.0015	0.0006 ± 0.0011
Den sites and random points at 4000‐m scale		
Distance to road (m)	878 ± 913.1	797 ± 918.8
Elevation (m)	2190 ± 329.7	2134 ± 380.6
Tree cover (%)	29 ± 27.1	27 ± 27.0
Slope (°)	19 ± 8.2	15 ± 8.3
Aspect (°)	156 ± 95.2	161 ± 103.6
Ruggedness	0.0011 ± 0.0015	0.0005 ± 0.0009

*Note*: Dens were identified within the eastern Sierra Nevada and western Great Basin from 2011 to 2022. Field collected variables were measured from August to December 2022.

**TABLE 2:** Effect sizes and Bayesian credible intervals for time series survival models on the effect of snow, number of consecutive days of sub‐freezing temperatures (≤−10°C minimum temperature; entrance model only), number of consecutive days of above‐freezing temperatures (>0°C minimum temperature; exit model only), elevation, and sex (reference class: male) on the daily probability of den entrance and exit for black bears.

Variable	Effect estimate	CI lower limit	CI upper limit
Entrance			
Ordinal date	0.0254	0.00085	0.04909
Entry snow	0.0539	−0.5999	0.6199
Consec. freeze days	0.2710	−0.1597	0.6603
Elevation	−0.2587	−0.4968	−0.0143
Sex (female effect)	0.2390	−0.2948	0.7764
Exit			
Ordinal date	0.0591	0.0423	0.0769
Exit snow	−0.1313	−0.4518	0.1904
Consec. warm days	0.7475	0.1204	1.3149
“Sex” by “Warm Days' interaction”	−0.5314	−1.4982	0.4488
Elevation	−0.1260	−0.4918	0.2432
Sex (female effect)	−0.7916	−1.8984	0.2358

*Note*: Dens of GPS radio‐collared black bears were identified within the eastern Sierra Nevada and western Great Basin from 2011 to 2022. Environmental data were obtained from NRCS SNOTEL sites.

In addition to measuring characteristics in the field at 26 den sites, we measured geospatial variables at the full set of 116 dens. Each of the 116 den sites was paired with randomly located points that were generated using ArcGIS (Esri, Redlands, CA, USA). Those random points were generated within three different buffer zones around the den: a 300‐, a 1000‐, and a 4000‐m radius. This paired design was modified from Pigeon, Cote, and Stenhouse (2016) who used random points within 1500 m of each den site and Bard and Cain III (2020) who used random points within 50–250 m from each den site. We included a third spatial scale at 4000 m based on previous work that has shown habitat selection for both black and grizzly bears to be influenced by features up to 4000 m away (Beckmann et al., 2015; Mattson et al., 1986). Using those three different buffer zones allowed us to investigate selection at both local and broad spatial scales (Gray et al., 2017).

To ensure that we were appropriately characterizing availability from each of our buffer zones, we tested our models with iteratively increasing numbers of random points paired with each den site. For the 300‐m scale model containing field collected data, which were paired with geospatial data, we used two random sites per den site because of the constraints of field work. After graphing the model coefficients for each variable at each increasing number of random points, we determined that coefficients stabilized at 80 random points for the 300‐ and 1000‐m buffer zones, and at 150 random points for the 4000‐m buffer zone, and therefore used each of those numbers of random points in our final models containing just geospatial variables. Our method of pairing den sites with random points at different spatial scales resulted in four different datasets for analysis: 26 dens with both field measured characteristics and geospatial variables paired with two random sites at 300 m, 116 den sites with only geospatial variables paired with 80 random sites at 300 m, 116 dens with only geospatial variables paired with 80 random sites at 1000 m, and 116 dens with geospatial variables paired with 150 random sites at 4000 m.

We extracted geospatial variables including values of slope, tree cover, elevation, distance to the nearest road, aspect, and terrain ruggedness at each den site and random location (Table S1, Table 1). Slope, elevation, and aspect were extracted from a digital elevation model (30‐m resolution). Tree cover (30‐m resolution) and distance from each den and random location to the nearest road (30‐m resolution) were both acquired from the Western USA Geodatabase at the University of Nevada Reno. Our estimate of ruggedness is a modified version of the vector ruggedness model (30‐m resolution) (Dilts et al., 2023; Sappington et al., 2007).

To investigate entrance and exit dates based on environmental covariates, we obtained environmental data from Snowpack Telemetry (SNOTEL) sites managed by the USDA Natural Resources Conservation Service (NCRS) National Water and Climate Center. We chose four SNOTEL automated data collection sites, Mt. Rose (Site 652), Heavenly (Site 518), Marlette Lake (Site 615), and Tahoe City (Site 809), as being representative of the den‐site locations in our study (mean distance between the den and the nearest SNOTEL site was 9.2 ± 6.49 km); and for each environmental covariate, we used mean values of the four sites at each day (Figure 1). Entrance date was determined by NDOW biologists as the date where collar locations become stationary in the winter, or the last transmission of that collar for the winter. Exit date was determined by NDOW biologists as the date that the bear left the den site and continued traveling away from the site for two consecutive GPS locations without returning. Some den sites did not have entrance and exit dates recorded, so we used a subset of dens for each analysis: entrance date (*n* = 68) and exit date (*n* = 42). We also excluded dens that were too far from a SNOTEL location to document snow depth or temperature. For example, we excluded den sites from the Pine Nut Mountains and Virginia Range in the western Great Basin because there are no SNOTEL sites in those ranges. Our chosen environmental covariates were minimum daily temperature and daily snow depth. We also included elevation in the entrance and exit models because of differences in amounts of snow accumulation by elevation, which was extracted from ArcGIS. In order to test a window of time that would be ecologically meaningful based on the likely time frame that bears were preparing to enter dens, we averaged the daily snow depths of the 2 weeks preceding entrance or exit for each den site (Gonzalez‐Bernardo, Russo, et al., 2020). To determine the effects of temperature on entrance and exit from dens, we used the number of consecutive nights below freezing for entrance and above freezing for exit.

### 2.3 Statistical analysis

We began all analyses by standardizing variables using a z‐transformation (Zar, 2010). We transformed aspect, which is a circular variable, using both a sine (east–west) and a cosine (north–south) transformation, prior to standardizing (McKee et al., 2015; Zar, 2010). We then assessed collinearity for all predictor variables using a Pearson correlation matrix in R (4.2.2 R Core Team, 2022) and based on biological relevance, eliminated one of any two variables in the same model that were highly correlated |*r*| > .70 with one another (Heffelfinger et al., 2020; Long et al., 2014; Stewart et al., 2002). In our analysis, no variables were highly correlated. Since we were interested in investigating multiple scales of selection, we analyzed each of the four datasets separately, resulting in four separate models: (1) both landscape characteristics and characteristics measured in the field compared with two random points close to the den (300‐m), (2) landscape characteristics collected from geospatial data compared to 80 random points close to the den (300‐m), (3) landscape characteristics compared with 80 random points at a middle distance from the den (1000‐m), and (4) landscape characteristics compared with 150 random points at a far distance from the den (4000‐m).

To quantify characteristics of den sites selected by bears, we used conditional logistic regression using the “clogit” function in the survival package in R version 4.2.2 (R Core Team, 2022; Therneau, 2022; Therneau & Grambsch, 2000). Conditional logistic regression allowed us to use a matched‐case control format for used and available (random) locations (Bard & Cain III, 2020; Manly et al., 2002; Schafer et al., 2018). We used the “stepAIC” function in the MASS package in R (Venables & Ripley, 2002) to rank models by Akaike's Information Criterion corrected for small sample size (AICc; Burnham & Anderson, 2002) and to identify a top‐performing model. We then evaluated our top‐performing model for uninformative effect sizes defined as those with 95% intervals that crossed zero (Arnold, 2010). Because of small sample sizes, we were unable to include den type in our analyses, but we included interactions with covariates with sex to address differences in selection of den sites between males and females.

We assessed differences in types of dens between females and males using a Fisher's Exact Test (Zar, 2010). We then used a series of two‐sample *z*‐tests for proportions on each type of den to look for differences between males and females within each den type. For the *z*‐tests, we adjusted α from 0.05 to 0.01 by dividing 0.05 by the number of *z*‐tests performed (5) using a Bonferroni correction for repeated comparisons (Zar, 2010). We tested for regional differences in den locations and types between the Sierra Nevada (Carson Range) and the Great Basin (the Virginia Range and the Pine Nut Mountains) using Fisher's Exact Test and a series of two‐sample *z*‐tests.

We used a two‐sample *t*‐test to test for differences in den entry and exit dates based on sex, after converting entry and exit dates to ordinal dates and adjusted entry dates for those that overlapped January 1 (Zar, 2010). We used one‐way analysis of variance (ANOVA) on means of den entry and exit dates to test for differences among years (Zar, 2010). In 2013 and 2018, there were no exit dates recorded, so we were unable to include those years in the analysis.

To test the effect of static and time‐varying environmental conditions on individual dates of entrance and exit from den, we fit denning chronology models (analogous to known‐fate survival models) in a Bayesian framework using JAGS (Just Another Gibbs Sampler) (Plummer, 2003). In the “entrance” model, bears entered den with a daily probability *p*_*ent*_, and in the “exit” model, bears emerged from den with a daily probability *p*_*exit*_. Both *p*_*ent*_ and *p*_*exit*_ were modeled as a logit‐linear function of covariates including elevation, snowpack, consecutive days with sub‐freezing temperatures, day of year, and sex (see below for details). Entry and exit from den in the exit and entrance models, respectively, was assumed to represent an absorbing state (e.g., once a bear had entered den in the “entrance” model, we assumed it could not exit). Because we were interested in time periods two weeks before beginning hibernation, we quantified the number of consecutive sub‐freezing days (≤−10°C) prior to each potential entrance date (“consecutive freeze days” for modeling the timing of entrance to den) and the number of consecutive above‐freezing days (>0°C) prior to each potential exit date (“consecutive warm days” for modeling the timing of exit from den). Similarly, we used the SNOTEL data to quantify the mean daily snow depth across the 2 weeks before each potential entrance and exit date (Table S1). We also included the (static) den elevation in these models because of variation in temperature and snow depth by elevation. Because we hypothesized differences in overall timing patterns between female and male bears, we included sex as a covariate in both the entrance and exit models (males were treated as the reference class). Finally, we tested for an interaction between sex and the number of consecutive freeze days (entrance model) or warm days (exit model). To visualize the effects of covariates (e.g., elevation, number of consecutive sub‐freezing days) on denning chronology, we used our models to derive the median date of entry and exit (50% quantile of the distribution of entry or exit dates) across specific gradients (holding all other covariates at their mean values).

We assigned uninformative uniform priors (min = 0, max = 1) on all probabilities and weakly regularized Gaussian priors (mean = 0, var = 10) to each regression coefficient. We ran each model with three chains and 10,000 iterations, and assessed convergence using visual assessment of trace plots and ensuring all potential scale reduction factors (R‐hat) were below 1.1 (Brooks & Gelman, 1998).

## 3 RESULTS

**FIGURE 2:**
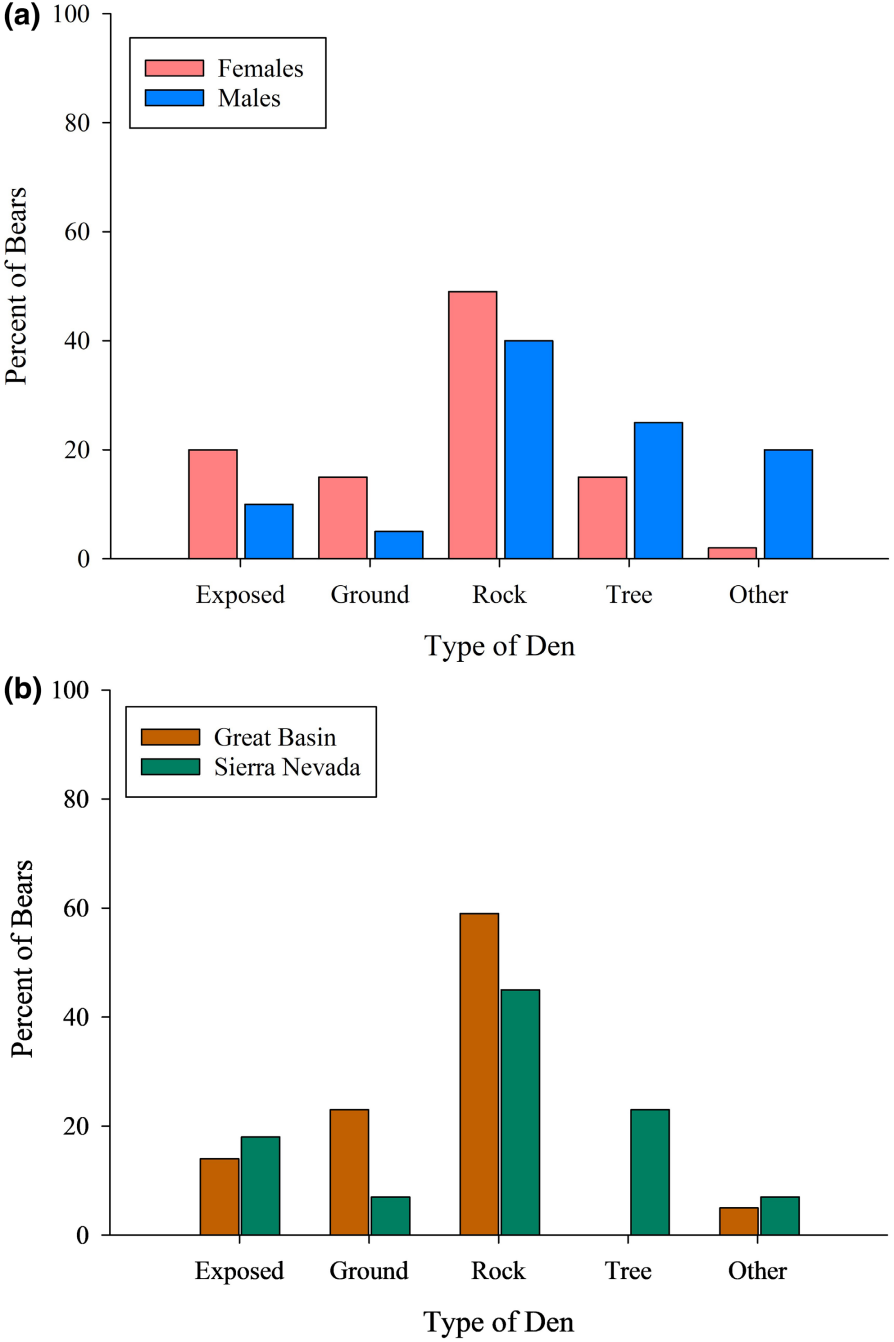
Percentage of black bears occupying each recorded den type by sex (a) and study region (b) in the eastern Sierra Nevada and western Great Basin (2011–2022). Total den sites were 15.4% exposed dens, 46.2% rock dens, 11.5% excavated ground dens, 16.7% tree dens, and 10.3% other (*n* = 76). Two‐sample *z*‐tests indicated no difference between the proportion of males and females using any type of den. Our alpha (α) level was adjusted to 0.01 based on the Bonferroni correction: Exposed dens (*p =* .52), ground dens (*p =* .48), tree dens (*p =* .46), rock dens (*p =* .61), and other (*p =* .02). Two‐sample *z*‐tests indicated no difference between the proportion of bears in the Sierra Nevada and Great Basin using any type of den. Exposed dens (*p =* 1), ground dens (*p =* .09), tree dens (*p =* .04), rock dens (*p =* .60), and other (*p =* 1).

Our hypothesis that females and males differed overall in types of dens used was weakly supported (Fisher's exact test *p* = .045); however, no pairwise comparisons (*z*‐proportion tests) were interpretable after applying a Bonferroni correction (revised α of 0.01) (Figure 2). We also investigated proportional differences in use of den types by bears between the Sierra Nevada and Great Basin study areas, and found that bears differed in types of dens used (Fisher's exact test *p* = .036) (Figure 2). Again, no pairwise comparisons were interpretable based on α = 0.01, although bears appeared to use tree dens more in the Sierra Nevada (*n* = 13) than in the Great Basin (*n* = 0, *p =* .04) (Figure 2).

**FIGURE 3:**
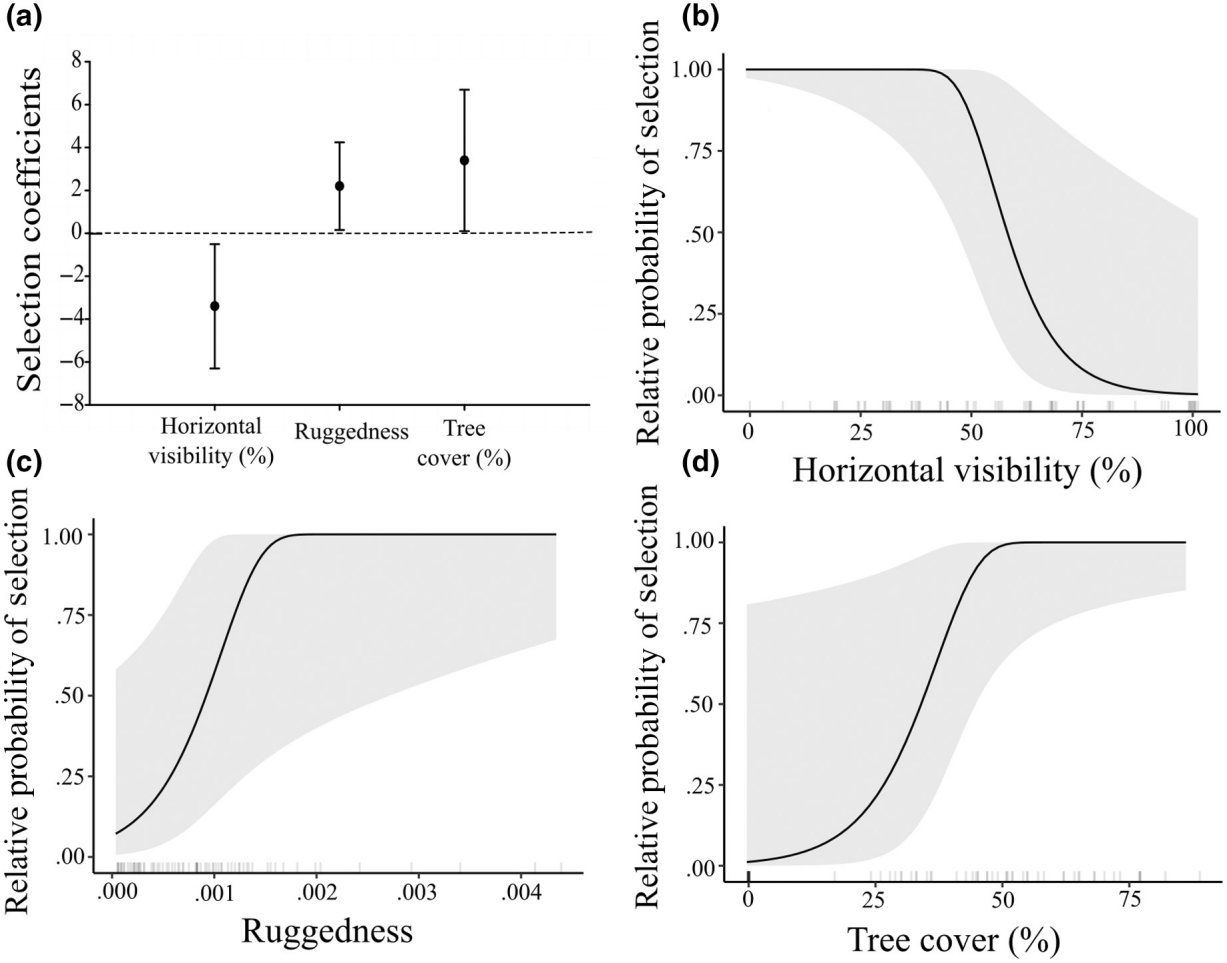
Effect plots illustrating the functional relationship between the relative probability of den‐site selection (*Y*‐axis) and the three covariates included in the best performing (based on AICc) conditional logistic regression model for selection of black bear den sites identified within the eastern Sierra Nevada and western Great Basin from 2011 to 2022 (*n* = 26). Effect sizes combining geospatial and field collected characteristics (a). Curves of the relative probability of selection for horizontal visibility (b), tree cover (c), and terrain ruggedness (d) with 95% confidence intervals.

**FIGURE 4:**
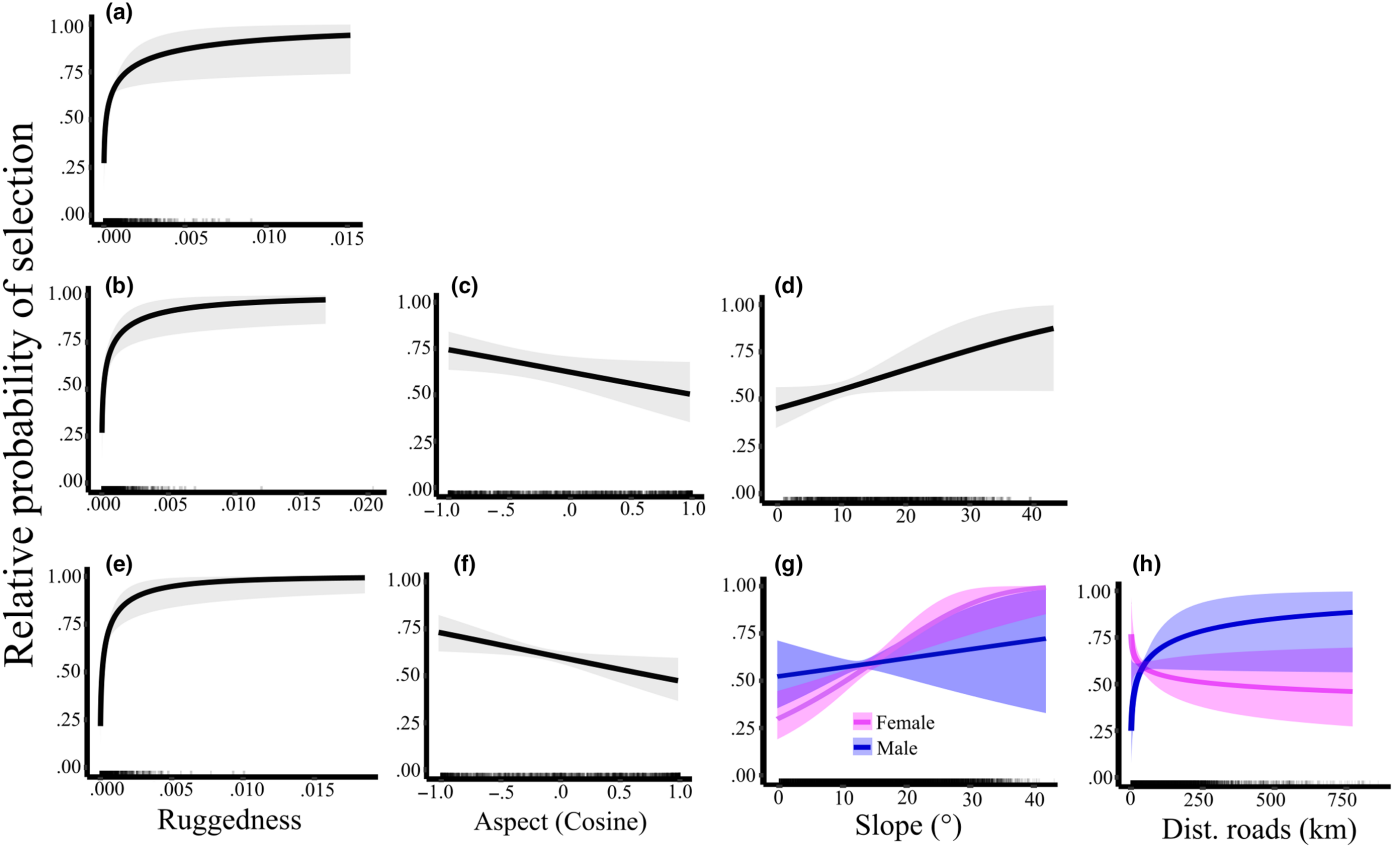
Curves of the relative probability of selection using conditional logistic regression for selection of black bear den sites in the eastern Sierra Nevada (2011–2022) (*n* = 116) using landscape‐level geospatial characteristics. Spatial scales are: 300‐m buffer with 80 random points per den (a), 1000‐m buffer with 80 random points per den (b–d), and 4000‐m buffer with 150 random points per den (e–h). Only in the 4000‐m buffered area were interactions of sex with slope and distance to roads detected.

The top‐performing model at 300‐m scale using remotely sensed data for site selection of all dens with geospatial landscape‐level variables (*n* = 116 used, 9280 available) included only ruggedness (*β* = 0.368, 95% CI [0.105 to 0.631] Table 1); note that this and following coefficient estimates are derived from centered and scaled covariates and are modeled on the logit scale, indicating selection for rugged terrain for placement of dens. For our 300‐m‐scale models that included both geospatial and field collected data (*n* = 25 used, 50 available), the top‐performing model included horizontal visibility (*β* = 3.38, 95% CI [0.15 to 0.661]), ruggedness (*β* = 2.19, 95% CI [0.19 to 4.19]), and tree cover (*β* = −3,44, 95% CI [−6.33 to −0.504]), suggesting avoidance of high visibility areas and selection for rugged terrain with high tree cover for placement of the den (Table 1, Figure 3). Probability of selection of den sites at 300‐m scale declined with increasing horizontal visibility above ~50% (Figure 3). Probability of selection of den site was greatest in rugged terrain with more than ~40% tree cover (Figure 3). At the 1000 m scale (*n* = 116 used, 9280 available), the top‐performing model included ruggedness (*β* = 0.415, 95% CI [0.182 to 0.647]), slope (*β* = 0.231, 95% CI [−0.005 to 0.467]), and northness (−0.225, 95% CI [0.447 to −0.0023]) (Figure 4). At the 4000‐m scale (*n* = 116 used, 17,400 available), our top‐performing model included slope(*β* = 0.510, 95% CI [0.233 to 0.805]), distance to road (*β* = −0.171, 95% CI [−0.465 to 0.124]), ruggedness (*β* =0.510, 95% CI [0.283 to 0.738]), northness (*β* = −0.239, 95% CI [−0.438 to −0.039]), an interaction between slope and sex (*β* = −0.411, 95% CI [−0.840 to 0.018]), and an interaction between distance to road and sex (*β* = 0.571, 95% CI [0.044 to 1.097]) (Figure 4). Females selected den sites on steeper slopes and closer to roads than did males.

**FIGURE 5:**
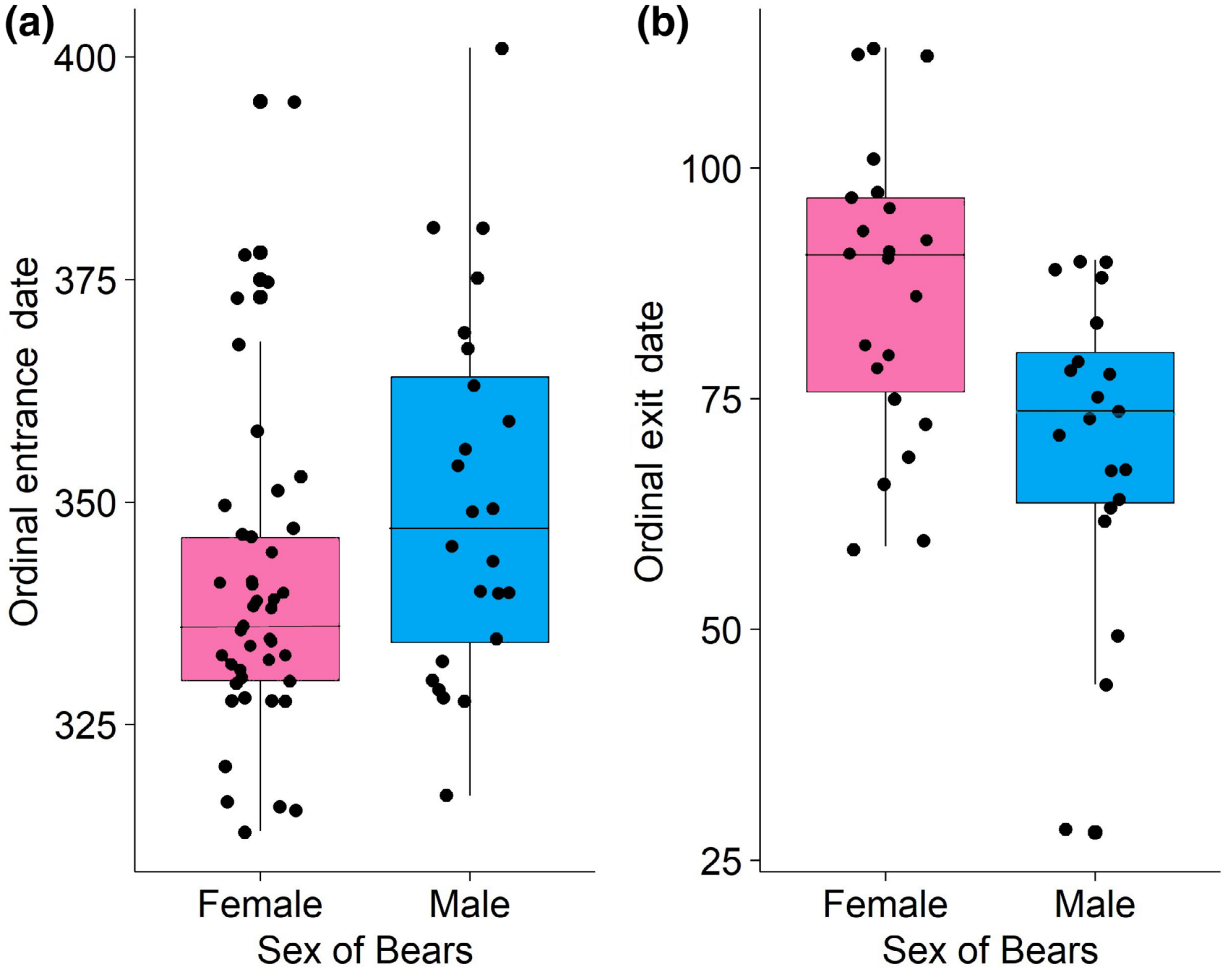
Ordinal date of entry (a) or exit (b) for male and female black bears in the eastern Sierra Nevada and western Great Basin from 2011 to 2022 (*n* = 70). of entrance was adjusted for entrance dates after January 1. Mean date of entry was December 10th for all individuals across all years (2011–2022). Mean date of exit was March 20th for all individuals across all years. Two‐sample *t*‐tests support that females enter dens earlier (*p =* .04) and exit later (*p =* .002) than males.

Included in the study were 70 individuals that had recorded either exit or entrance date from the den. In general, females entered dens earlier (*p =* .04) and exited later (*p =* .002) than did males (Figure 5). The mean date of entry for all bears across all years was December 6th (±17) ($\bar{x}$ ± SD) for females and December 16th (±18) for males. The mean date of exit for all bears across all years was March 28th (±16) for females and March 12th (±13) for males. When testing for differences among years, there was no difference in entry date for females (*p =* .7) or males (*p =* .6) among years (2013–2022) (Figure 5). There was also no difference in exit date for females (*p =* .6) or males (*p =* .8) among years (2014–2022, excluding 2018) (Figure 5).

**FIGURE 6:**
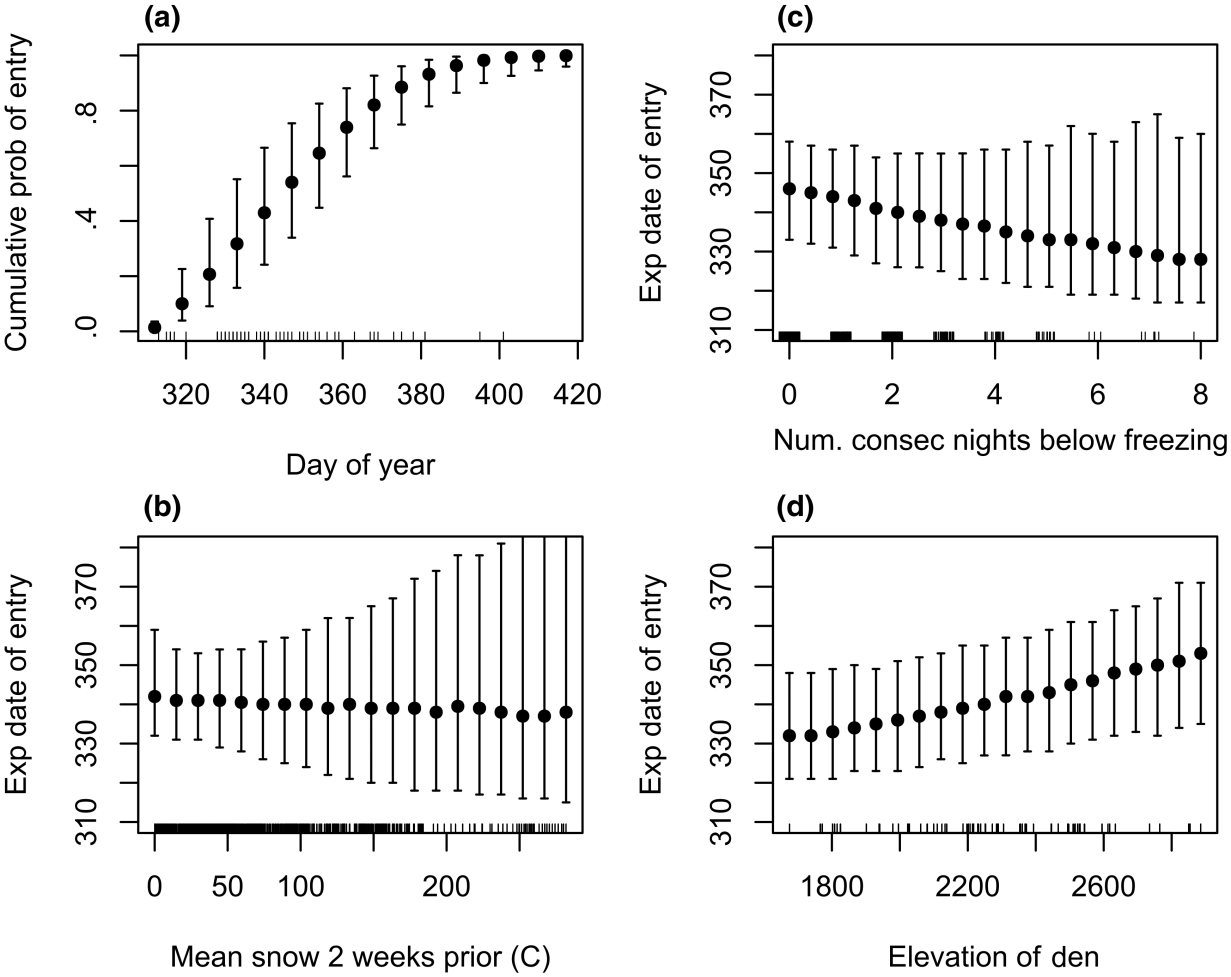
Cumulative probability of entry into den for each ordinal date during autumn for black bear dens in the eastern Sierra Nevada (2011–2022) (*n* = 68) based on a time series survival model (a). Mean snow depth 2 weeks prior to entrance (b), expected date of entry based on cumulative number of consecutive days below freezing (−5°C) prior to entrance (c), and elevation of den site (d).

**FIGURE 7:**
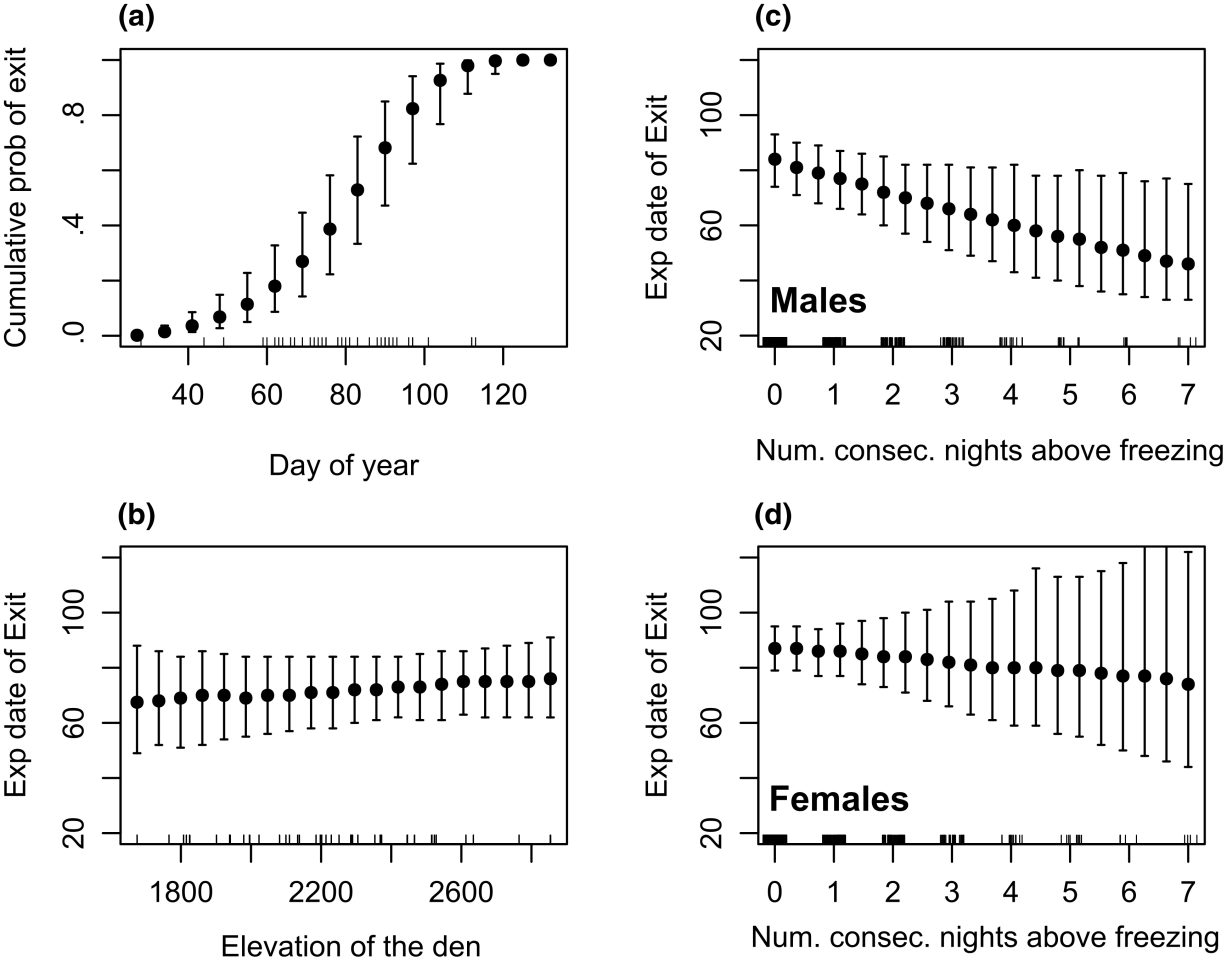
Cumulative probability of exit for number of days and nights above freezing for each ordinal date during spring for black bear dens (*n* = 42) in the eastern Sierra Nevada (2011–2022) based on a time series survival model (a). Expected date of exit based on day of the year, cumulative numbers of days above freezing 10 days prior to exit (b), elevation of the den (c), and elevation of den site (d). Mean snowpack had a very small effect size in this model and is not visualized here.

Our results indicated a weak influence of the number of consecutive sub‐freezing days on the daily probability of den entrance (and consequently on the median date of den entrance; Table 2, Figure 6), and a strong influence of the number of consecutive warm (above‐freezing) days on the daily probability of den exit (Table 2), with strong consequences for the median date of exit; (Figures 6 and 7). We detected no temperature‐by‐sex interaction on the probability of den entrance, but there was evidence for a temperature‐by‐sex interaction on the probability of den exit, with males more responsive to the number of consecutive above‐freezing days than females (Figure 7, expected consequences for the median date of entry illustrated in this figure). Mean snowpack was not strongly predictive for either den entrance or exit (Table 2). Elevation was weakly predictive of both the probability of den entrance and the probability of den exit (Table 2; Figures 6 and 7).

## 4 DISCUSSION

Bears occupied dens predominantly under rocks and within hollow trees, reflecting selection for hidden and protected dens. A large rock or tree would obscure much or all of the hibernating bear within the den cavity, suggesting that protection from disturbance was an important factor in choosing a den site. Although, Waller et al. (2012) observed bears using mostly tree and ground dens, bears in our study occupied some ground and exposed dens, but rock piles in both study areas and hollow trees in the Sierra Nevada were much more commonly used by bears in our study. An enclosed den may also offer environmental protection with stability in temperature and humidity that would not be present in an exposed or ground den. Pinyon pine and juniper trees, characteristic of the Western Great Basin, are smaller and less likely to be available as hollow trees for bears to use as dens compared to the large species of pines that are common in the Carson Range. We observed no tree dens used by bears in the Great Basin, and we were unable to document the presence of any hollow trees that could have been occupied as dens by black bears. Black bears inhabiting that region used primarily rock dens, which were readily available in that area. Goodrich and Berger (1994) reported a similar difference, recording 16 tree dens in the Carson Range in the Sierra Nevada, but only two in the Sweetwater Range in the Great Basin.

Spatial scale strongly influenced selection of den sites, and our model results varied with spatial scale, with the exception that ruggedness of terrain entered every one of our models. At all three spatial scales, including the 300‐m model that consisted of both field collected and geospatial data, we observed dens to be located in more rugged terrain than was generally available. At the 1000‐ and 4000‐m scales, den sites were located on steeper slopes and more northerly aspects than was available. Although the ruggedness metric is calculated from both slope and aspect, it is not correlated with those covariates (Dilts et al., 2023; Sappington et al., 2007). In the 300‐m scale analysis that included field collected and remotely sensed data, characteristics that had the most influence on selection of den sites were canopy cover of trees, low horizontal visibility (e.g., higher concealment), and ruggedness of terrain, which supported our hypothesis that bears selected for high concealment of their dens. Interestingly, for the models that only included remotely sensed data, each spatial scale resulted in the addition of one or more characteristics with the 300‐m model having only ruggedness in the model.

Waller et al. (2012) have noted that evaluation selection of den sites is a hierarchical process and should be examined at multiple spatial scales to improve understanding of how bears select den sites. Additionally, factors that have the greatest effect on fitness in terms of selection should occur at greater spatial scales and lesser fitness limiting factors should occur at smaller spatial scales (McLoughlin et al., 2002; Rettie & Messier, 2000; Waller et al., 2012). Nevertheless, characteristics immediately around the den, such as concealment, and the structure and type of den affect energy efficiency and concealment, which also have direct effects on fitness, through survival of offspring and body condition of individuals over winter (Baldwin & Bender, 2008; Shiratsuru et al., 2020). Our data indicated that including field‐based measurements with the remotely sensed data at the smallest scale resulted in a model that showed strong selection for concealment of the den site in addition to being located in rugged terrain, which was the only variable that entered the remotely sensed model at that scale. Therefore, we suggest that at localized spatial scales including data collected in the field combined with remotely sensed data resulted in more effective understanding of selection of den sites. As we expanded the spatial scale that we modeled selection, the models became somewhat more complex. Ruggedness of the terrain in the area of a den site is likely the most important characteristic used by bears to select a den site since that characteristic entered both the field collected and all of the remotely sensed models. Ruggedness may add protection and concealment of the den site to reduce disturbance for hibernating bears (Hellgren & Vaughan, 1989; Hightower et al., 2002; Waller et al., 2012). Disturbance of bears in winter dens has been identified as a concern for den abandonment in our study system (Goodrich & Berger, 1994).

At the broad 4000‐m scale, we detected interactions between sex with slope and sex with distance to roads. Females selected den sites on steeper slopes and closer to roads than did males. Excavated dens on slopes under 25° have been suggested to decrease thermal effectiveness of the den, in addition to increasing the risk of snow falling into the den opening or flooding in the spring (Crupi et al., 2020; Servheen & Klaver, 1983). The majority of dens used in our study were in hollow trees or under rocks, which provided more protection and may be less dependent on the effects of slope compared with excavated dens. Distance to the nearest road could influence disturbance, since dens that are further away from roads would be less likely to be disturbed by motor vehicles; males selected den sites farther from roads possibly to avoid those disturbances (Gantchoff et al., 2019; Lustig et al., 2021). Gantchoff et al. (2019) also observed female black bears denning closer to roads than males and suggested that this behavior by females, especially those with young, may be a way to reduce the risk of infanticide by males. We were not able to differentiate nonpregnant, pregnant, or females that were accompanied by dependent young, and although other studies have noted that avoidance of males was less likely to be observed in nonpregnant females (Gantchoff et al., 2019) the signal was strong in our data.

One of the challenges of this study was that we used random sites to determine availability rather than “potential den sites.” Humans perceive the world differently than do animals (Nams et al., 2006), and attempting to define a den site would lead to a much larger source of bias than using randomly generated locations. Therefore, we were unable to include den types in our models, and we also were unable to quantify availability of dens across the landscape. Bears in our study areas used multiple types of dens, including trees, rocks, exposed, or ground dens, and as a result almost any location could be a potential den site.

We observed almost no fidelity to or reuse of dens and saw only one instance of a den being reused, which was by a different individual. Similarly, other studies have reported few instances of individuals reusing dens, and den reuse is usually made by different individuals (Klenzedorf et al., 2002; LeCount, 1983; Linnell et al., 2000). Klenzedorf et al. (2002) not only reported some fidelity to den type in West Virginia, but also reported only a few instances of individuals reusing dens. Fidelity to a den was either absent or not recorded in many black bear studies, although there is some evidence of fidelity to den sites in areas with low availability of suitable dens (Davis et al., 2012). There is some evidence of fidelity to the same general area in brown bear populations (Sorum et al., 2019). Fidelity to a den has been hypothesized as an adaptation to low availability of existing den sites (Alt & Gruttadauria, 1984; Davis et al., 2012; Johnson & Pelton, 1980), which is unlikely to be a constraint in our study areas because there is an abundance of large trees and rock piles (Grayson, 2011). The lack of fidelity to dens that we observed suggests that suitable den sites are not limiting for bears in our study areas. Waller et al. (2012) noted that reuse of dens in the Southeast is typically in tree dens (Crook & Chamberlain, 2010; Linnell et al., 2000). Tree dens may be strongly selected in areas prone to flooding (Schwartz et al., 1987), which is not a selective factor in our arid study areas.

Similar to other studies of black bears, females occupied dens longer than males; both entering earlier and exiting later (Fowler et al., 2019; Mitchell et al., 2005; Waller et al., 2012), although that pattern did not occur in New Mexico (Inman et al., 2007). Timing of exit from dens was strongly related to the cumulative number of days with minimum temperatures above freezing for males, but that relationship was weak for females. Miller et al. (2017) reported that spring temperatures were negatively correlated with exit data and that when spring temperatures were lower, exit from dens was delayed. Doan‐Crider and Hellgren (1996) suggested that emergence from dens by females was probably more closely related to development and growth of young than to food availability or weather conditions, which may explain why weather was less likely to affect exit of dens by females. Others have reported that reproductive state of females (not pregnant, pregnant, with cubs, or with yearlings) affected timing of exit and entrance to dens (Gantchoff et al., 2019; Immell et al., 2013; Johnson & Pelton, 1980). An important caveat to our analysis of den entrance and exit dates is that we were unable to include reproductive status of females or age of individuals, which has been shown to have as much if not more influence than environmental characteristics (Johnson et al., 2017). Pregnant female bears have also been seen to enter dens earlier than nonpregnant females (Fowler et al., 2019; Garshelis et al., 2020), thus lack of those data may be confounding our results. Timing of entrance and exit also has been tied to body condition, and bears in good body condition may den earlier than those in poor condition (Schooley et al., 1994). We were unable to adequately quantify body condition of bears at the timing of entrance, nor were we able to document food availability, or to tie environmental conditions to potential food availability, but that could be an area of further research with this population of bears.

Our results regarding selection of den sites reinforce previous work, which reported that habitat selection varied relative to local versus landscape‐level characteristics (Crook & Chamberlain, 2010; Reynolds‐Hogland et al., 2007). Nevertheless, our work provides some unique findings not shown in previous work. Our examination of selection at multiple spatial scales illuminates the importance of rugged terrain in selection of den sites. Additionally, cumulative days above freezing strongly affected timing of exit of dens for males, although that effect was weaker for females. Nevertheless, there may still be a knowledge gap in linking den‐site selection specifically to reproductive fitness. Additionally, changes in the length and severity of winter affect food availability, which if connected to timing of den entrance and exit, could change bear behavior. Human encroachment into habitat could further affect selection of den sites with the potential of restricting availability of or access to den sites in wildland areas.

## AUTHOR CONTRIBUTIONS

**Morgan E. Long:** Formal analysis (equal); investigation (equal); methodology (equal); writing – original draft (lead). **Kelley M. Stewart:** Conceptualization (equal); funding acquisition (lead); investigation (equal); methodology (equal); project administration (lead); supervision (lead); writing – original draft (supporting); writing – review and editing (lead). **Heather Reich:** Conceptualization (equal); data curation (equal); investigation (equal); methodology (equal); supervision (supporting); writing – review and editing (equal). **Carl W. Lackey:** Conceptualization (equal); data curation (equal); investigation (equal); resources (equal); writing – review and editing (equal). **Jon P. Beckmann:** Conceptualization (equal); data curation (equal); investigation (equal); methodology (equal); writing – review and editing (equal). **Kevin T. Shoemaker:** Formal analysis (lead); investigation (equal); methodology (equal); writing – review and editing (equal).

## CONFLICT OF INTEREST STATEMENT

The authors have no conflicts of interest or competing interests.

## Supporting information


Data S1:

## Data Availability

Data are available upon request from Nevada Department of Wildlife.

## References

[ece311689-bib-0001] Alt, G. L. , & Gruttadauria, J. M. (1984). Reuse of black bear dens in northeastern Pennsylvania. Journal of Wildlife Management, 48, 236–239.

[ece311689-bib-0002] Amspacher, K. M. , Jiménez, F. A. , & Nielsen, C. K. (2023). Winter denning behavior of striped skunks and interspecific den activity a their dens: Implications for pathogen transmission. Wildlife Research, 50, 160–168. 10.1071/WR22002

[ece311689-bib-0003] Anderson, M. , Derocher, A. E. , Oystein, W. , & Aars, J. (2012). Polar bear (*Ursus maritimus*) maternity den distribution in Svalbard, Norway. Polar Biology, 35, 499–508. 10.1007/s00300-011-1094-y

[ece311689-bib-0004] Andreasen, A. M. , Stewart, K. M. , Longland, W. S. , & Beckmann, J. P. (2021). Prey specialization by cougars on feral horses in a desert environment. The Journal of Wildlife Management, 85(6), 1104–1120. 10.1002/jwmg.22087

[ece311689-bib-0005] Arnold, T. W. (2010). Uninformative parameters and model selection using Akaike's information criterion. Journal of Wildlife Management, 74, 1175–1178. 10.2193/2009-367

[ece311689-bib-0006] Baldwin, R. A. , & Bender, L. C. (2008). Den‐site characteristics of black bears in Rocky Mountain National Park, Colorado. Journal of Wildlife Management, 72(8), 1717–1724. 10.2193/2007-393

[ece311689-bib-0007] Baldwin, R. A. , & Bender, L. C. (2010). Denning chronology of black bears in eastern Rocky Mountain National Park, Colorado. Western North American Naturalist, 70, 48–54.

[ece311689-bib-0008] Bard, S. M. , & Cain, J. W., III . (2020). Investigation of bed and den site selection by American black bears (*Ursus americanus*) in a landscape impacted by forest restoration treatments and wildfires. Forest Ecology and Management, 460, 117904. 10.1016/j.foreco.2020.117904

[ece311689-bib-0009] Beckmann, J. P. , & Berger, J. (2003). Rapid ecological and behavioral changes in carnivores: The responses of black bears (*Ursus americanus*) to altered food. Journal of Zoology, 261, 207–212. 10.1017/S0952836903004126

[ece311689-bib-0010] Beckmann, J. P. , Waits, L. P. , Hurt, A. , Whitelaw, A. , & Bergen, S. (2015). Using detection dogs and RSPF models to assess habitat suitability of bears in greater Yellowstone. Western North American Naturalist, 75(4), 396–405. 10.3398/064.075.0410

[ece311689-bib-0011] Beecham, J. J. , Reynolds, D. G. , & Hornocker, M. G. (1983). Black bear denning activities and den characteristics in west‐central Idaho. International Conference on Bear Research and Management, 5, 79–86. 10.2307/3872522

[ece311689-bib-0012] Berger, J. (2004). The last mile: How to sustain long‐distance migration in mammals. Conservation Biology, 18, 320–331.

[ece311689-bib-0013] Bieber, C. , Juškaitis, R. , Turbill, C. , & Ruf, T. (2012). High survival during hibernation affects onset and timing of reproduction. Oecologia, 169, 155–166. 22095523 10.1007/s00442-011-2194-7

[ece311689-bib-0014] Boutros, D. , Breitenmoser‐Wursten, C. , Zimmermann, F. , Ryser, A. , Molinari‐Jobin, A. , Capt, S. , Guntert, M. , & Breitenmoser, U. (2007). Characterisation of Eurasian lynx *Lynx lynx* den sites and kitten survival. Wildlife Biology, 13, 417–429.

[ece311689-bib-0015] Brooks, S. P. , & Gelman, A. (1998). General methods for monitoring convergence of iterative simulations. Journal of Computational and Graphical Statistics, 7(4), 434–455.

[ece311689-bib-0016] Burnham, K. P. , & Anderson, D. R. (2002). Model selection and multimodel inference: A practical information‐theoretic approach (2nd ed.). Springer‐Verlag.

[ece311689-bib-0017] Crook, A. C. , & Chamberlain, M. J. (2010). A Mulitscale assessment of Den selection by Black bears in Louisiana. The Journal of Wildlife Management, 74(8), 1639–1647. 10.2193/2009-31

[ece311689-bib-0018] Crupi, A. P. , Gregovich, D. P. , & White, K. S. (2020). Steep and deep: Terrain and climate factors explain brown bear (*Ursus arctos*) alpine den site selection to guide heli‐skiing management. PLoS ONE, 15(9), e0238711. 10.1371/journal.pone.0238711 32966287 PMC7511016

[ece311689-bib-0019] Davis, H. , Hamilton, A. N. , Harestad, A. S. , & Weir, R. D. (2012). Longevity and reuse of Black bear dens in managed forests of coastal British Columbia. The Journal of Wildlife Management, 76(3), 523–527. 10.1002/jwmg.253

[ece311689-bib-0020] Dilts, T. E. , Blum, M. E. , Shoemaker, K. T. , Weisberg, P. J. , & Stewart, K. M. (2023). Topographic ruggedness indices in ecology: Past, present, and future. Landscape Ecology, 38, 1395–1410. 10.1007/s10980-023-01646-6

[ece311689-bib-0021] Doan‐Crider, D. L. , & Hellgren, E. C. (1996). Population characteristics and winter ecology of black bears in Coahuila, Mexico. Journal of Wildlife Management, 60, 398–407.

[ece311689-bib-0022] Fowler, N. L. , Belant, J. L. , Wang, G. , & Leopold, B. D. (2019). Ecological plasticity of denning chronology by American black bears and brown bears. Global Ecology and Conservation, 20, e00750. 10.1016/j.gecco.2019.e00750

[ece311689-bib-0023] Fowler, N. L. , Spady, T. J. , Wang, G. , Leopold, B. D. , & Belant, J. L. (2021). Denning, metabolic suppression, and the realization of ecological opportunities in Ursidae. Mammal Review, 51, 465–481. 10.1111/mam.12246

[ece311689-bib-0024] Friebe, A. , Evans, A. L. , Arnemo, J. M. , Blanc, S. , Brunberg, S. , Fleissner, G. , Swenson, J. E. , & Zedrosser, A. (2014). Factors affecting date of implantation, parturition, and Den entry estimated from activity and body temperature in free‐ranging Brown bears. PLoS ONE, 9(7), e101410. 10.1371/journal.pone.0101410 24988486 PMC4079694

[ece311689-bib-0025] Friebe, A. , Swenson, J. E. , & Sandegren, F. (2001). Denning chronology of female brown bears in Central Sweden. Ursus, 12, 37–45.

[ece311689-bib-0026] Fuglei, E. , & Ims, R. A. (2008). Global warming and effects on the arctic fox. Science Progress, 91, 175–191. 18717368 10.3184/003685008X327468 PMC10361153

[ece311689-bib-0027] Gantchoff, M. G. , Beyer, D. , & Belant, J. L. (2019). Reproductive class influences risk tolerance during denning and spring for American black bears (*Ursus americanus*). Ecosphere, 10(4), e02705. 10.1002/ecs2.2705

[ece311689-bib-0028] Garshelis, D. L. , Noyce, K. V. , Ditmer, M. A. , Coy, P. L. , Tri, A. N. , Laske, T. G. , & Iaizzo, P. A. (2020). Remarkable adaptations of the American Black bear help explain why it is the most common bear: A long‐term study from the center of its range. In V. Penteriani & M. Melletti (Eds.), Bears of the world (pp. 53–62). Cambridge University Press. 10.1017/9781108692571.006

[ece311689-bib-0029] Gonzalez‐Bernardo, E. , Giulia, B. , Mar Delgado, M. , & Vincenzo, P. (2020). The role of spring temperatures in the den exit of female brown bears with cubs in southwest Europe. Ursus, 31(13), 1–11. 10.2192/URSUS-D-19-00015.1

[ece311689-bib-0030] Gonzalez‐Bernardo, E. , Russo, L. F. , Valderrabano, E. , Fernandez, A. , & Penteriani, V. (2020). Denning in brown bears. Ecology and Evolution, 10, 6844–6862. 10.1002/ece3.6372 32724555 PMC7381752

[ece311689-bib-0031] Goodrich, J. M. , & Berger, J. (1994). Winter recreation and hibernation Black bears *Ursus americanus* . Biological Conservation, 67, 105–110.

[ece311689-bib-0032] Gray, C. , Hooker, M. , & Chamberlain, M. (2017). Reproductive and denning ecology of the Central Georgia American black bear population. Ursus, 27(2), 67–77. 10.2192/URSU-D-16-00009.1

[ece311689-bib-0033] Grayson, D. K. (2011). The Great Basin: A natural prehistory. University of California Press.

[ece311689-bib-0034] Hayes, F. E. (1995). Definitions for migrant birds: What is a neotropical migrant? The Auk, 112, 521–523.

[ece311689-bib-0035] Heffelfinger, L. J. , Stewart, K. M. , Shoemaker, K. T. , Darby, N. W. , & Bleich, V. C. (2020). Balancing current and future reproductive investment: Variation in resource selection during stages of reproduction in a Long‐lived herbivore. Frontiers in Ecology and Evolution, 8, 163. 10.3389/fevo.2020.00163

[ece311689-bib-0036] Hellgren, E. C. (1998). Physiology of hibernation in bears. Ursus, 10, 467–477.

[ece311689-bib-0037] Hellgren, E. C. , & Vaughan, M. R. (1989). Denning ecology of black bears in a southeastern wetland. Journal of Wildlife Management, 53, 347–353.

[ece311689-bib-0038] Hightower, D. A. , Wagner, R. O. , & Pace, R. M., III . (2002). Denning ecology of female American black bears in south central Louisiana. Ursus, 13, 11–17.

[ece311689-bib-0039] Humphries, M. M. , Thomas, D. W. , & Kramer, D. L. (2003). The role of energy availability in mammalian hibernation: A cost–benefit approach. Physiological and Biochemical Zoology, 76, 165–179. 10.1086/367950 12794670

[ece311689-bib-0040] Immell, D. , Jackson, D. W. H. , & Boulay, M. C. (2013). Denning ecology of American black bears in the Cascade Mountains of western Oregon. Ursus, 24(1), 1–12.

[ece311689-bib-0041] Inman, R. M. , Costello, C. M. , Jones, D. E. , Inman, K. H. , Thompson, B. C. , & Quigley, H. B. (2007). Denning chronology and design of effective bear management units. Journal of Wildlife Management, 71, 1476–1483.

[ece311689-bib-0042] Johnson, H. E. , Lewis, D. L. , Verzuh, T. L. , Wallace, C. F. , Much, R. M. , Willmarth, L. K. , & Breck, S. W. (2017). Human development and climate affect hibernation in a large carnivore with implications for human‐carnivore conflicts. Journal of Applied Ecology, 55, 663–672. 10.1111/1365-2656.13820

[ece311689-bib-0043] Johnson, K. G. , & Pelton, M. R. (1980). Environmental relationships and the denning period of black bears in Tennessee. Journal of Mammalogy, 61, 653–660.

[ece311689-bib-0044] Klenzedorf, S. A. , Vaughan, M. R. , & Martin, D. D. (2002). Den‐type use and fidelity of American Black bears in Western Virginia. Ursus, 13, 39–44.

[ece311689-bib-0045] Krofel, M. , Špacapan, M. , & Jerina, K. (2017). Winter sleep with room service: Denning behaviour of brown bears with access to anthropogenic food. Journal of Zoology, 302, 8–14.

[ece311689-bib-0046] Lackey, C. W. , Beckmann, J. P. , & Sedinger, J. (2013). Bear historical ranges revisited: Documenting the increase of a once‐extirpated population in Nevada. Journal of Wildlife Management, 77(4), 812–820. 10.1002/jwmg.548

[ece311689-bib-0047] Laurenson, M. K. (1994). High juvenile mortality in cheetahs and its consequences for maternal care. Journal of Zoology (London), 234, 387–408.

[ece311689-bib-0048] LeCount, A. L. (1983). Denning ecology of black bears in central Arizona. Bears: Their Biology and Management: International Association for Bear Research and Management, 5, 71–78.

[ece311689-bib-0049] Lemon, P. E. (1956). A spherical Densiometer for estimating Forest overstory density. Forest Science, 2, 314–320.

[ece311689-bib-0050] Libel, N. S. , Belant, J. L. , Leopold, B. D. , & Owen, P. A. (2011). Despotism and risk of infanticide influence grizzly bear den‐site selection. PLoS ONE, 6, e24133. 21935378 10.1371/journal.pone.0024133 PMC3173359

[ece311689-bib-0051] Linnell, J. D. C. , Swenson, J. E. , Andersen, R. , & Barnes, B. (2000). How vulnerable are denning bears to disturbance. Wildlife Society Bulletin, 28(2), 400–413.

[ece311689-bib-0052] Long, R. A. , Bowyer, T. R. , Porter, W. P. , Mathewson, P. , Monteith, K. L. , & Kie, J. G. (2014). Behavior and nutritional condition buffer a large‐bodied endotherm against direct and indirect effects of climate. Ecological Monographs, 84(3), 513–532. 10.1890/13-1273.1

[ece311689-bib-0053] López‐Alfaro, C. , Robbins, C. T. , Zedrosser, A. , & Nielsen, S. E. (2013). Energetics of hibernation and reproductive trade‐offs in brown bears. Ecological Modelling, 270, 1–10. 10.1016/j.ecolmode1.2013.09.002

[ece311689-bib-0054] Lustig, E. , Bales, L. S. , Leslie, D. , Luttbeg, B. , & Fairbanks, S. (2021). Resource selection by recolonizing American Black bears. The Journal of Wildlife Management, 85(3), 531–542. 10.1002/jwmg.22010

[ece311689-bib-0055] Manly, B. F. J. , McDonald, L. L. , Thomas, D. L. , McDonald, T. L. , & Erickson, W. P. (2002). Resource selection by animals: Statistical design and analysis for field studies (2nd ed.). Kluwer Academic Publishers.

[ece311689-bib-0056] Mattson, D. J. , Knight, R. R. , & Blanchard, B. M. (1986). The effects of developments and primary roads on grizzly bear habitat use in Yellowstone National Park, Wyoming. International Conference on Bear Research and Management, 7, 259–273. 10.2307/3872633

[ece311689-bib-0057] McKee, C. J. , Stewart, K. M. , Sedinger, J. S. , Bush, A. P. , Darby, N. W. , Hughson, D. L. , & Bleich, V. C. (2015). Spatial distributions and resource selection by mule deer in an arid environment: Responses to provision of water. Journal of Arid Environments, 122, 76–84. 10.1016/j.jaridenv.2015.06.008

[ece311689-bib-0058] McLoughlin, P. D. , Case, R. L. , Gau, R. J. , Cluff, H. D. , Mulders, R. , & Messier, F. (2002). Hierarchial habitat selection by barren ground grizzly bears in the central Canadian Arctic. Oecologia, 132, 102–108. 28547280 10.1007/s00442-002-0941-5

[ece311689-bib-0059] Melvin, R. G. , & Andrews, M. T. (2009). Torpor induction in mammals: Recent discoveries fueling new ideas. Trends in Endocrinology and Metabolism, 20(10), 490–498. 19864159 10.1016/j.tem.2009.09.005 PMC2788021

[ece311689-bib-0060] Miller, J. A. , Smith, T. S. , Auger, J. , Black, H. L. , & Allphin, L. (2017). The late‐denning activities of American black bear in Utah. Ursus, 27, 78–89.

[ece311689-bib-0061] Mitchell, F. S. , Onorato, D. P. , Hellgren, E. C. , Skiles, J. R., Jr. , & Harveson, L. A. (2005). Winter ecology of American black bears in a desert montane Island. Wildlife Society Bulletin, 33, 164–171.

[ece311689-bib-0062] Nams, V. O. , Mowat, G. , & Panian, M. A. (2006). Determining the spatial scale for conservation purposes – An example with grizzly bears. Biological Conservation, 128, 109–119.

[ece311689-bib-0063] Nelson, R. A. , Folk, E. G. , Pfeiffer, E. W. , Craighead, J. J. , Jonkel, C. J. , & Steiger, D. L. (1980). Behavior, biochemistry, and hibernation in Black, grizzly, and polar bears. Bears: Biology and their Management, 5, 284–290.

[ece311689-bib-0064] Noyce, K. V. , & Garshelis, D. L. (1994). Body size and blood characteristics as indicators of condition and reproductive performance in Black bears. International Conference on Bear Research and Management, 9(1), 481–496.

[ece311689-bib-0065] Ordiz, A. , Stoen, O. G. , Langebro, L. , Brunberg, S. , & Swenson, J. (2009). A practical method for measuring horizontal cover. Ursus, 20(2), 109–113. 10.2192/08SC031.1

[ece311689-bib-0066] Pelton, M. R. , Beeman, L. E. , & Eagar, D. C. (1980). Den selection by black bears in the great Smokey Mountains National Park. Bears: Their Biology and Management: International Association for Bear Research and Management, 4, 149–151. 10.2307/3808099

[ece311689-bib-0067] Pigeon, K. E. , Cote, S. D. , & Stenhouse, G. B. (2016). Assessing Den selection and Den characteristics of grizzly bears. The Journal of Wildlife Management, 80(5), 884–893. 10.1002/jwmg.1069

[ece311689-bib-0068] Pigeon, K. E. , Stenhouse, G. B. , & Cote, S. D. (2016). Drivers of hibernation: Linking food and weather to denning behavior of grizzly bears. Behavioral Ecology and Sociobiology, 70, 1745–1754. 10.1007/s00265-016-2180-5

[ece311689-bib-0069] Plummer, M. (2003). JAGS: A program for analysis of Bayesian graphical models using Gibbs sampling . Proceedings of the 3rd international workshop on distributed statistical computing (DSC 2003), Vienna, 20–22 March 2003, 1–10.

[ece311689-bib-0070] R Core Team . (2022). R: A language and environment for statistical computing. R Foundation for Statistical Computing. https://www.R‐project.org/

[ece311689-bib-0071] Rettie, W. J. , & Messier, F. (2000). Hierarchical habitat selection by woodland caribou: Its relationship to limiting factors. Ecography, 23, 466–478.

[ece311689-bib-0072] Reynolds‐Hogland, M. J. , Mitchell, M. S. , Powell, R. A. , & Brown, D. C. (2007). Selection of Den sites by Black bears in the southern Appalachians. Journal Of Mammology, 88(4), 1062–1073. 10.1644/06-MAMM-A-329R1.1

[ece311689-bib-0073] Robitaille, J.‐F. , Proulx, G. , & Do Linh San, E. (2020). On the use of den and resting site terminology for species in the Martes complex. Canadian Wildlife Biology and Management, 9, 2.

[ece311689-bib-0074] Ryan, C. W. , & Vaughan, M. R. (2004). Den characteristics of Black bears in southwestern Virginia. Southeastern Naturalist, 3, 569–668.

[ece311689-bib-0075] Sappington, M. J. , Longshore, K. M. , & Thompson, D. B. (2007). Quantifying landscape ruggedness for animal habitat analysis: A Case study using Bighorn sheep in the Mojave Desert. The Journal of Wildlife Management, 71(5), 1419–1426. 10.2193/2005-723

[ece311689-bib-0076] Schafer, T. L. J. , Breck, S. W. , Baruch‐Mordo, S. , Lewis, D. L. , Wilson, K. R. , Mao, J. S. , & Day, T. L. (2018). American black bear den‐site selection and characteristics in an urban environment. Ursus, 29(1), 25–31. 10.2192/URSUS-D-17-00004.2

[ece311689-bib-0077] Schooley, R. L. , McLaughlin, C. R. , Matula, G. J. , & Krohn, W. B. (1994). Denning chronology of female Black bears: Effects of food, weather, and reproduction. Journal Of Mammology, 75(2), 466–477.

[ece311689-bib-0078] Schwartz, C. C. , Miller, S. D. , & Franzmann, A. W. (1987). Denning ecology of three black bear populations in Alaska. Bears: Their biology and management. International Association for Bear Research and Management, 7, 281–291.

[ece311689-bib-0079] Servheen, C. , & Klaver, R. (1983). Grizzly bear dens and denning activity in the mission and Rattlesnake Mountains, Montana . Bears: Their Biology and Management, Vol. 5, A Selection of Papers from the Fifth International Conference on Bear Research and Management. 201‐207.

[ece311689-bib-0080] Shiratsuru, S. , Friebe, A. , Swenson, J. E. , & Zedrosser, A. (2020). Room without a view – Den excavation in relation to body size in brown bears. Ecology and Evolution, 10, 8044–8054. 10.1002/ece3.6371 32788960 PMC7417226

[ece311689-bib-0081] Sorum, M. S. , Joly, K. , Wells, A. G. , Cameron, M. D. , Hilderbrand, G. V. , & Gustine, D. D. (2019). Den‐site characteristics and selection by brown bears (*Ursus arctos*) in the central Brooks Range of Alaska. Ecosphere, 10(8), e02822. 10.1002/ecs2.2822

[ece311689-bib-0082] Stewart, K. M. , Bowyer, T. R. , Kie, J. G. , Cimon, N. J. , & Johnson, B. K. (2002). Temporospatial distribution of elk, mule deer, and cattle: Resource partitioning and competitive displacement. Journal Of Mammology, 83(1), 229–244. 10.1644/1545-1542(2002)083<0229:TDOEMD>2.0.CO;2

[ece311689-bib-0083] Therneau, T. (2022). A package for survival analysis in R . R package version 3.4–0, https://CRAN.R‐project.org/package=survival

[ece311689-bib-0084] Therneau, T. , & Grambsch, P. (2000). Modeling survival data: Extending the cox model. Springer.

[ece311689-bib-0085] USDA, NRCS . (2023). The PLANTS Database. National Plant Data Team. Retrieved March 22, 2023, from http://plants.usda.gov

[ece311689-bib-0086] van Manen, J. T. , Lackey, C. W. , Beckmann, J. P. , Muller, L. I. , & Li, Z. H. (2019). Assimilated diet patterns of American black bears in the Sierra Nevada and western Great Basin, Nevada, USA. Ursus, 30e3, 40–50. 10.2192/URSUS-D-17-00031.2

[ece311689-bib-0087] Venables, W. N. , & Ripley, B. D. (2002). Modern applied statistics with S (4th ed., p. 498). Springer Science+Business Media.

[ece311689-bib-0088] Waller, B. , Belant, J. , Young, B. , Leopold, B. , & Simek, S. (2012). Denning chronology and den characteristics of American black bears in Mississippi. Ursus, 23(1), 6–11. 10.2307/41818966

[ece311689-bib-0089] Wynn‐Grant, R. , Ginsburg, J. , Lackey, C. , Sterling, E. , & Beckmann, J. P. (2018). Risky business: Modeling mortality risk near the urban‐wildland interface for a large carnivore. Global Ecology and Conservation, 16, e00443. 10.1016/j.gecco.2018.e00443

[ece311689-bib-0090] Zar, J. H. (2010). Biostatistical analysis (5th ed.). Prentice Hall.

