# Supplementary material for: Selection of den sites and chronology of denning by black bears in the eastern Sierra Nevada and western Great Basin
Source: Ecol Evol. 2024 Jul 10;14(7):e11689. doi: 10.1002/ece3.11689 (PMC11236437; doi:10.1002/ece3.11689)
Supplement: Supplementary file 1 — Data S1: [file ECE3-14-e11689-s001.zip › draft_metadata.docx]

**File: mean_weather_data**

Data Collection:

Data was pulled from the NRCS SNOTEL database (add link?) using the ____ tool. Covariates are averaged across four SNOTEL sites: Mt. Rose (Site 652), Heavenly (Site 518), Marlette Lake (Site 615), and Tahoe City (Site 809)

Data Columns:

Date: date of observation

Mean_tempC: the mean temperature in Celsius for each date, averaged across all four SNOTEL sites

Min_tempC: the minimum temperature in Celsius for each date, averaged across all four SNOTEL sites

Wateryr_cm: the cumulative amount of precipitation in centimeters from the start of the Water Year (October 1) to the date recorded, averaged across all four SNOTEL sites

Precip_cm: the incremental amount of precipitation in centimeters that fell each day, averaged across all four SNOTEL sites

Snow_cm: the recorded snow depth in centimeters on each date, averaged across all four SNOTEL sites

**File: den_entrance_data**

Data Collection:

ID, sex, date, and location (not included in data) were determined by the Nevada Department of Wildlife.

Data Columns:

ID: unique ID of each bear

Sex: sex of bear, 0 = male, 1 = female

Entry_year: year that the bear entered the den

Julian_entrance: ordinal date that the bear entered the den; adjusted for the start of the year (ie: ordinal dates that were past January 1 were added to 365)

Elev: elevation in meters of each den site. Elevation was estimated from a 1-m DEM within ArcMap.

**File: den_exit_data**

Data Collection:

ID, sex, date, and location (not included in data) were determined by the Nevada Department of Wildlife. Elevation was estimated from a 1-m DEM within ArcMap.

Data Columns:

ID: unique ID of each bear

Sex: sex of bear

Exit_year: year that the bear exited the den

Julian_exit: ordinal date that the bear exited the den

Elev: elevation in meters of each den site

**Files: 300m_sites, 1000m_sites, 4,000m_sites**

Note: All three files have the same columns; the difference is the buffer zone that random points were calculated in. So, the dataset contains the same den sites with randomly generated points within a 300m scale, a 1,000m scale, and a 4,000m scale in order to be compared to den sites in a used vs available framework.

Data Collection:

Den locations were identified by the Nevada Department of Wildlife (coordinates not included in data). Random points were generated in ArcGIS, and geospatial characteristics were estimated in ArcGIS.

Data Columns:

ID: unique ID of each bear

Den_Type: whether the measured site is a den site or a randomly generated point, 0 = random point, 1 = den site

CID: variable that links used and random dens for analyses.

Treecover: tree cover (%) in 30-m resolution

Dist_to_water: distance (m) to the nearest water source at 90m resolution

Dist_to_road: distance (m) to the nearest main or forest service road (excludes non-motorized trails)

Slope: slope in degrees estimated from digital elevation model at 30-m resolution

Ruggedness: measurement of terrain ruggedness at 30-m resolution

Aspect: measure of direction the slope is facing: both sine and cosine transformed at 30-m resolution

Elevation: elevation estimated from digital elevation model at 30-m resolution

**File: fieldwork_data**

Data Collection: Den locations were identified by the Nevada Department of Wildlife (coordinates not included in data). Random points were generated in ArcGIS, and geospatial characteristics were estimated in ArcGIS. For field work collected variables; all measures were taken within a 15-m circular plot around the den or random site.

Data Columns:

Bear_ID: unique ID of each bear

Den_type: whether the measured site is a den site or a randomly generated point, 0 = random point, 1 = den site

Dist_to_roads: distance to the nearest main or forest service road (excludes non-motorized trails)

Slope: slope in degrees estimated from digital elevation model at 30-m resolution

Treecover: percent tree cover in 30-m resolution

Elevation: elevation estimated from digital elevation model at 30-m resolution

Aspect: measure of direction the slope is facing: both sine and cosine transformed at 30-m resolution

Dist_to_water: distance in meters to the nearest water source at 90m resolution

Vrml_30m: same as “rugg” above, measurement of terrain ruggedness at 30-m resolution

bare_ground_pct: Percent of plot absent of ground cover measured visually (field collected)

shrubs_pct: percent of ground cover within the plot covered by shrubs (field collected)

trees_pct: percent of ground cover within the plot covered by trees (field collected)

other_pct: percent of ground cover within the plot covered by other substrates (field collected)

Num_trees: the number of trees found within each plot (field collected)

Horizontal_vis: percent of the den site or center of random plot that is visible 7.5 m away at a height of 1 m (field collected)

Min_dist: minimum distance of total concealment, the minimum distance from the center of the plot that either the den site or center of random plot that is not visible at all (field collected)

Canopy_cover: a separate measure from “treecover”; this is the canopy cover measured from the center of each plot using a spherical densitometer (field collected)
