# Supplementary material for: Selection of den sites and chronology of denning by black bears in the eastern Sierra Nevada and western Great Basin
Source: Ecol Evol. 2024 Jul 10;14(7):e11689. doi: 10.1002/ece3.11689 (PMC11236437; doi:10.1002/ece3.11689)
Supplement: Supplementary file 1 — Data S1: [file ECE3-14-e11689-s001.zip › Long et al. Supplement Table 1.docx]

SELECTION OF DEN SITES AND CHRONOLOGY OF DENNING BY BLACK BEARS IN THE EASTERN SIERRA NEVADA AND WESTERN GREAT BASIN

Morgan E. Long, Kelley M. Stewart, Kevin T. Shoemaker, Heather Reich, Carl W. Lackey, Jon P. Beckmann

**M. E. Long, K. M. Stewart, and K. T. Shoemaker**, Ecology Evolution and Conservation Biology Graduate Program and Department of Natural Resources and Environmental Science, University of Nevada, 1664 N. Virginia St MS186, Reno, Reno, NV 89557

**H. Reich and C. W. Lackey**, Nevada Department of Wildlife, 6980 Sierra Center Parkway #120, Reno, NV 89511

**J. P. Beckmann**, Wildlife Conservation Society, 212 S Wallace Ave., Suite 101, Bozeman, MT 59715;

Current Address: Kansas Department of Wildlife and Parks, 512 SE 25^th^ Ave, Pratt, KS 67124

Corresponding author: Kelley M. Stewart

Address: Natural Resources and Environmental Science

1664 N. Virginia St, Mail Stop 186

University of Nevada, Reno

Reno, NV 89557

Tel: (775) 313-5280

Email: [KelleyS@unr.edu](mailto:KelleyS@unr.edu)

**Supplement Table 1.** Definitions of model terms for analysis of black bear den site selection at three spatial scales, and for environmental covariates used to test influence of weather on date of entrance into dens and date of exit out of dens for black bears. Dens of GPS radio-collared black bears were identified within the eastern Sierra Nevada and western Great Basin from 2011-2022. Environmental covariates were collected from four SNOTEL (Snow Telemetry) sites in the eastern Sierra Nevada.

|  | Variable Name | Variable | Definition |
| --- | --- | --- | --- |
| *Den Site Selection Model Terms* | | | |
|  | DistRoad | Distance to road (m) | distance to the nearest main or forest service road (excludes non-motorized trails) |
|  | Elevation | Elevation (m) | elevation estimated from digital elevation model at 30-m resolution |
|  | HV | Horizontal visibility (%) | percent of the den site or center of random point that is visible 7.5 m away at a height of 1 m |
|  | Tree | Tree cover (%) | percent tree cover in 30-m resolution |
|  | Slope | Slope (°) | slope in degrees estimated from digital elevation model at 30-m resolution |
|  | Aspect | Aspect (°) | measure of direction the slope is facing: both sine and cosine transformed at 30-m resolution |
|  | BG | Bare ground (%) | Percent of plot absent of ground cover measured visually |
|  | Ruggedness | Ruggedness | measurement of terrain ruggedness at 30-m resolution |
|  | | | |
| *Environmental Characteristics* | | | |
|  | Entry temperature | Number of consecutive days below freezing prior to entry (° C) | Number of consecutive days below freezing (-5° C) prior to entry into the den. |
|  | Entry snow | Mean entry snow depth (cm) | Mean daily snow depth of the two weeks prior to each entry date (cm) |
|  | Exit temperature | Minimum temperature above freezing prior to exit (°C) | Number of consecutive days above freezing (0° C) for 10 days prior to exit date. |
|  | Exit snow | Mean exit snow depth (cm) | Mean daily snow depth of the two weeks prior to each exit date (cm) |
|  | Elevation | Elevation (m) | elevation estimated from digital elevation model at 30-m resolution |
